# On the General Causes of Chronic Diseases, Particularly Pulmonary Phthisis, and on the Means of Preventing These Affections, &c.

**Published:** 1845-07

**Authors:** 


### ART. VII.

*Causes générales des Maladies Chroniques, spécialement de la Phthisie Pulmonaire, et Moyens de prévenir le Développement de ces Affections, &c.* Par A. FOURCAULT, de l'Académie Royale de Médecine.—*Paris,* 1844.

*On the general Causes of Chronic Diseases, particularly Pulmonary Phthisis, and on the Means of preventing these Affections, &c.* By A. FOURCAULT, of the Royal Academy of Medicine, &c.—*Paris,* 1844. 8vo, pp. 480.

THE volume before us consists of a number of essays, the common bond of their union being the relations of the cutaneous function to pathological states, and especially to that constituting phthisis pulmonalis and the numerous class of disorders known as scrofulous. M. Fourcault's more general proposition is, that the causes of disease are not so diversified as commonly supposed, that they exercise their power principally upon the skin, and that they act in general by diminishing or suppressing the cutaneous exhalation or insensible transpiration. If it be suppressed suddenly, diseases follow of an acute character; if slowly, they take the chronic form.

A moist atmosphere diminishes the cutaneous transpiration and consequently induces disease. In France, in small towns containing about 2000 inhabitants, and situate so that they enjoy a dry air, the mortality from phthisis ranges from 1 in 40 to 1 in 60. Persons may live in a moist atmosphere with impunity if they favour the cutaneous transpiration by active exercise. That is to say, the employments that induce sweat are little favorable to scrofula or consumption, although carried on even in water. The most usual disease of artisans of this class is rheumatism, to which tanners, wool-washers, laundresses, dyers, &c. are subject. Miners work in damp, ill-ventilated localities, yet they are rarely phthisical, the warmth of the mine and the severity of their labour affording a prophylaxis through the necessarily augmented perspiration. The following conclusion from these and other facts, as for instance, the health of animals confined in menageries, we give in the words of the author:

"These facts, which can be observed everywhere, prove conclusively that muscular exercise, either in the open air or in the house, is indispensable to health; that when man is deprived of it, he becomes liable to the most serious chronic maladies; for without it, the excreting function of the skin is inactive; the capillary circulation becomes languid, and a tendency to congestion of the viscera supervenes. Guided by these results of my first statistical researches, I have adopted this general principle: *To protect man or animals from tubercular affections, it is requisite that they be habitually exposed in the state of freedom to the influences of the atmosphere.*" (p. 24.)

Having deduced this principle, M. Fourcault proceeded on his travels, that he might further establish it by numerous observations. With this foregone conclusion he visited Holland, Belgium, the south of France, Italy, and England, and collected his facts in hospitals, almshouses, workhouses, prisons, factories, and agricultural colonies. He found that dissolute conduct, the action of dust on the air-passages, restricted positions, while at work, and tight-lacing have a secondary and unimportant influence in the development of phthisis pulmonalis. One example of this kind he derives from the hygienic condition of the *filles de pavé* at Paris. Parent-Duchatelet ascertained that they perish in great numbers if reclaimed, and kept closely to work with the needle. These repentant and reformed women die phthisical, or suffer from various chronic diseases. Those however who continue their debased occupation are fat, fresh-looking, and healthy, but they enjoy their liberty, take exercise, and are freely exposed to atmospheric vicissitudes.

An inquiry into the effects of sedentary professions generally shows that they co-operate with humidity in multiplying the number of chronic maladies. At Amsterdam and Leyden, for example, M. Fourcault found scrofula and phthisis extremely frequent. Of 457 deaths in the great hospital at Amsterdam during the year 1840, 101 were from phthisis, namely 66 males and 35 females. In Holland there are *ateliers de charité*, or charitable institutions, where the poor can have work, established both in the large towns, and in certain agricultural colonies founded for the special purpose of reclaiming waste and useless land. The mortality in the civic ateliers is much greater than in the rural: of 38 deaths in the latter, only 3 were from phthisis. The workhouses (dépôts de mendicité) present a high rate of mortality, although young and robust men are received.

The mortality varies from 1 in 9 to 1 in 6, the proportion in the dépôt de mendicité at Amsterdam. Analogous results are seen in France. This high mortality is the consequence, according to M. Fourcault's views, of deficient ventilation and exercise.

The seclusion and inactivity of a prison life is particularly favorable to the development of phthisis. At Vilvorde, out of 63 deaths, 34 were from phthisis. In the central prison at Ghent, of 168 deaths 87 were from phthisis. At Vorisy, during ten years, there were 282 deaths, 178 of which were from pulmonary consumption, and only 29 from acute disease. At Gaillon, the results are similar, but here dropsy and scorbutus are also fatal, the prisoners not being allowed animal food, or fermented drinks. At Auburn in the United States (a prison constructed for solitary confinement), 70 per cent. of the deaths are from phthisis.

The effects of sedentary employments in inducing phthisis are seen in the manufacturing town of Lille. Here the weavers, lacemakers, embroiderers, &c., die phthisical and scrofulous in great numbers. The general hospital there presents a remarkable proof of the fatal effects resulting from deficient exercise. The building is also an hospital or asylum for foundlings. The infants being received here are sent into the country, and on attaining a certain age are brought back to be educated. The girls are employed in spacious apartments at sedentary employments, the boys go out to follow different trades in the city. The latter free to go about and with ample scope for exercise are strong and robust; the former are pale, languid, and chlorotic. They seldom die of acute disease, but suffer from scrofulous affections and especially caries of the vertebræ. At Vienna, M. Fourcault found some mutilated mulberry trees opposite the windows of the girls' school-room in the foundling hospital there, and on inquiring the reason of their mutilation, was informed that their shade manifestly rendered the chronic affections from which the girls suffered more severe, and that since a freer evaporation and more light had been thus obtained, their health had visibly improved. As it was, a fifth of the females presented one or other form of rickets. At Marseilles, there is an asylum for orphans: in 21 years, 45 had died of pulmonary phthisis out of a total of 68 deaths.

M. Fourcault found the operatives of silk factories more liable to disease than those of cotton mills. The employment in mills generally is unhealthy in proportion as the rooms are narrow, dark, and crowded, the toil prolonged, and the labour light, or rather, not demanding much muscular effort. The inhalation of dust is much less injurious, he asserts, than is generally supposed. M. Fourcault quotes examples illustrative of this proposition. The contrary results are seen when the workrooms are spacious and well lighted and ventilated, as at Louviers and Elbœuf.

From these and similar facts M. Fourcault takes occasion to impress on his readers the great necessity of free exercise in the open air, so that the excreting functions of the skin shall be duly excited by muscular action and the surface itself exposed to a pure atmosphere. He quotes our own gracious Queen as an example in this respect to all persons whose circumstances will permit them to follow it. He also refers in a note to the hygienic treatment of the royal family of Belgium. Being at Ostend in 1840, he met the children walking on the sands in pursuance of the advice given by Sir James Clark and M. Lebeau.

*Phthisis in captive animals.* M. Fourcault devotes a short chapter to a consideration of the hygienic condition of captive animals. The facts are not different from those generally known in this country. It appears that in Paris the animals from tropical climates, and especially monkeys, usually die of tubercular disease. Tubercles are found in the liver, the bronchial and mesenteric glands, and even in the cellular tissue, as well as in the lungs.

An important fact illustrative of M. Fourcault's views has lately been observed in the garden of the Zoological Society of London. By freely ventilating the houses of the monkeys, &c., the great tendency to disease and death has been most happily interfered with. And it has been found that even tropical animals preserve their health much better when allowed to expose themselves in the open air even in winter, than when confined, as formerly, in close heated apartments; the good of the *exercise,* apparently, more than compensating for the evil of the lower temperature.

*Influence of humidity.* The injurious repression of the cutaneous excretion by moisture in the atmosphere has been already noticed. In this (the 4th) chapter, M. Fourcault enters more into detail. He observes that much still remains to be known, and much of what is known to be promulgated and understood. The controversies of Broussais and Laennec prove that they were both unacquainted with the important influence of suppressed perspiration in developing tubercles. He ridicules the proposition to send phthisical patients into marshy countries because it appears that phthisis is rare where intermittents prevail. For the same reason they might be sent into mountainous countries where acute diseases are most prevalent. The antagonism of phthisis and intermittents has been popularly known in England for many years. Even at the present moment, it is not unusual to have aged persons observe, that since there was less ague there has been more consumption. We think the apparently anomalous fact may be satisfactorily explained by the law of equilibrium between the causes of mortality referred to subsequently by M. Fourcault. The persons who formerly died of ague, or its sequelæ, would have died of consumption.

The effect of a moist locality on the numbers of deaths from phthisis is shown by some comparable instances. The village of Ezy is situate near the little town of Anet in the valley of the Eure. In the former, the deaths from phthisis constitute one eighth of the total deaths, in the latter they are only one fiftieth. Ezy is so situate, that to the west and north, it is sheltered by a mountain; to the east and south, by lofty trees which intercept the atmospheric currents, and so prevent evaporation. Anet, on the contrary, is situate on an elevated, open, sandy portion of the valley, accessible to the winds. Scrofula and other chronic diseases are most prevalent at Ezy, acute affections at Anet. The village of Fontenay-Saint-Père is made up of several hamlets differently situate. The people of those on an elevated site, and exposed to the south, are little subject to phthisis or scrofula, but the inhabitants of the low-lying damp hamlets are a prey to every form of scrofulous disease.

The influence of damp habitations and moist climates is also demonstrated by numerous facts, especial reference being made to England. Although one sixth of the deaths are from consumption, M. Fourcault thinks that disease is much less fatal than it would be were it not counteracted by our frequent sea-voyages, our "biftecks" and "rosbifs," and other salutary precautions.

*Humidity as the common cause of intermittents and phthisis.* M. Fourcault doubts the existence of a specific agent in the causation of intermittent fevers, and he thinks the hypothesis of an unknown poison unnecessary for the explanation of their phenomena. These fevers depend on the concurrence of three essential conditions, moisture of the air, elevation of temperature, and atmospheric vicissitudes. In all localities where these exist, sporadic or epidemic fevers will prevail, although there be no marshes; and he mentions several examples, as the mountains surrounding the Agro Romano at Tivoli, Subiac, Terni, &c. Again, in those years in which much rain falls, and a damp coldness at night follows suffocating heat during the day, epidemic fevers prevail; and, on the contrary, a year remarkable for a continued high and uniform temperature is remarkable for its healthiness. M. Fourcault is of opinion that the suppression of the cutaneous secretion after exercise, while the conditions mentioned are active, is the principal cause of intermittent fever; and he adduces such facts as he thinks conclusive on this point, but they appear to us to involve a petitio principii.

*The influence of physical agents on the development.* Dampness may not only produce the diseases already attributed to it, but may also alter the general conformation of man. In deep narrow valleys where the atmosphere is saturated with moisture, and its circulation impeded, goitre and cretinism, forms of disease closely allied to scrofula, are endemic. Leprosy, according to M. Fourcault, has a similar origin. These diseases are propagated hereditarily; if this fact be taken into estimate, it will be found that in proportion as the valleys widen, and the air (being more freely moved,) is drier, there endemic diseases become less frequent. To their residence in damp, dark huts, is to be attributed, M. Fourcault thinks, the stunted growth of the Esquimaux and Laplanders. Similar anomalies of development are found in those civilized communities in which individuals are exposed to similar morbific agencies, as weavers, miners, and others who work in damp, dark, and ill-ventilated places. The locality may even give the characteristics of a race; thus, the cradle of the Gael, with his stunted body and thick short limbs and fingers, has originally been in the winding valleys of high mountains, while the parent stock of the tall, well proportioned Cymri has enjoyed the sides of hills exposed freely to the sun and wind, or has resided on the sea-coast.

*Experiments on the functions of the skin.* This chapter is made up of an extract from the Report of the Commission on the Montyon Prize Essays in 1840, and which adjudged 2000 francs to M. Fourcault for his experiments. The original essay contained first, a series of experiments on animals, and secondly, deductions from those experiments. M. Fourcault conceived the idea of ascertaining the effect of suppressed cutaneous transpiration by varnishing or otherwise coating the whole or portions of the skin of living birds, dogs, &c., so that no moisture could escape. He found that changes in the characters of the blood and local lesions constantly followed a mechanical suppression of the cutaneous secretion. He ascertained that the skin has special relations to certain mucous membranes. A horse was impermeably coated over, and it died with a profuse discharge from the nose, and its blood resembled that of horses suffering from glanders. Several sheep so treated had a most violent coryza, and after death, inflammation of the mucous membranes of the nose was unequivocally manifest. In rabbits and dogs, diarrhea supervened and inflammation of the intestinal mucous membrane. In the latter, the liver was also enlarged, congested, and in a state of softening.

The lesions consequent on this mechanical suppression of cutaneous function were not confined to the mucous membranes; effusions into the serous sacs, as the pericardium and pleura, paraplegia, marasmus, and miliary tubercles in the lungs apparently of recent formation also resulted. When the whole body of the animal was coated over so that the air could not act on the skin, the respiration became difficult and laborious, and death quickly followed, often with convulsions. The veins and right cavities of the heart contained black blood forming soft diffluent clots, and large ecchymoses were seen in the lungs and other viscera. The capillaries were generally injected. A curious result followed the partial application of an impermeable coating. If for example, one half of the body only was covered, the capillaries on the inner surface of the skin of that half were found to be distended with a dark fluid blood, highly venous, while the capillaries of the skin not covered by the coating contained red blood, and much less of it. The line of demarcation between these two sets of capillaries could be readily traced. This is certainly an interesting fact, and the experiment merits repetition, as endermic medication may be much improved and rendered more precise by knowledge of this kind, not to mention the interesting neurological deductions to be made. M. Fourcault observed these phenomena in mammals, birds, and frogs. He experimented on the latter by suspending them by the feet in a vessel of oil, so that half the body should be immersed.

M. Fourcault attempted to ascertain the relations of the skin to the atmosphere, by placing animals in an exhausted receiver; permitting them, however, to take in air from without by the mouth. No other result followed than that the lungs, stomach, and intestines, were blown up with air from the want of atmospheric pressure. From experiments on the skin of the forearm, M. Fourcault is of opinion, that the cutaneous surface gives out no carbonic acid, and absorbs no oxygen. He has plunged mammals, birds, reptiles, and fishes into aerated water, and into water deprived of its air by boiling, and he has never found any perceptible difference in the duration of life of the animals thus treated. M. Fourcault thinks this experiment conclusive as to the non-respiring function of the skin. Comparative and transcendental physiology is so entirely opposed to this inference that there can be little doubt of some imperfection in the mode of conducting these experiments. It is well-established that frogs respire by the surface.

M. Fourcault mentions a solitary experiment on the function of the human skin, which is curious. When Leo X was elected pope, the age of gold he was to usher in was represented at Florence by a gilded living child; it soon died. M. Fourcault has gilded and silvered guinea-pigs, and they experienced exactly the same fate.

MM. Breschet and Becquerel repeated M. Fourcault's experiments, with special reference to the development of animal heat. They found that in half an hour after applying the varnish to a shaven rabbit, the temperature fell six degrees of Reaumur, and in half an hour more it had fallen seven additional degrees. M. Fourcault finds, however, that a diminution of temperature (as when guinea-pigs are placed in a damp cold atmosphere) diminishes the cutaneous transpiration, and even causes death. But if animals be kept in baths of oil or water, so that they can breathe and move freely, at a temperature of 15 to 20 degrees (of Reaumur) above the freezing point, although their warmth of body is not perceptibly diminished, they still die. The two fluids are not, however, fatal in equal times, as the animals die sooner in water than in oil; ducks perish in eight or nine hours in water, but will live several days in oil. M. Fourcault explains this, partly by supposing that water is a better conductor of heat than oil, and that in proportion as the temperature lowers the transpiration diminishes, and partly by the fact that the animals imbibe a considerable quantity. Birds thus treated before they die, vomit a large quantity of a clear and limpid fluid. The animal can be more readily restored however to life after immersion in water than in oil. Immersion in the latter induces the local lesions before described more decidedly, and these are the cause of death. The experiments detailed by M. Fourcault point out very clearly the necessity, in recovering persons drowned, of artificial warmth to the surface, and a free exposure of it to the air.

*The causes of albuminuria illustrated by experiments.* M. Fourcault being of opinion that albuminaria was a morbid result of suppression of the cutaneous function, instituted an examination of the urine of those animals whose surface he varnished or coated. He found that when dogs so treated began to exhibit symptoms of suffering and difficulty of breathing, the urine first became albuminous; the albumen being often mixed with blood-globules. Very generally when the animal succeeded in removing the substance with which it was coated, the albuminuria ceased, and the urinary salts reappeared in large quantities. A shaved rabbit was coated with dextrine, and so inclosed in an apparatus that the urine could be collected unmixed with feces. A considerable quantity of albumen appeared in the urine. In another rabbit so treated, the pericardium was found to contain an albuminous fluid. The urine of dogs thus coated, previously acid, became gradually less acid, then neutral, and when it contained a large quantity of albumen a tendency to alkalinity.

M. Fourcault flayed guinea-pigs and rabbits alive, replacing the skin in its proper position, and he was astonished to find that they lived two or three times longer than if they had been encased in an impermeable coating. They maintained their natural temperature and were lively and vigorous to within a few hours of their death. If, however, a layer of dextrine was laid over the flayed surface, albumen appeared. From these and other facts M. Fourcault infers that the skin is solely an excreting organ. Its function is to throw off the free lactic acid and lactates already present in the blood. If this acid be retained it is in excess, and, destroying the equilibrium of the organic affinities, precipitates albumen upon the urinary organs, when the soda of the urine renders it soluble. The cutaneous salts being also thrown back into the circulation pass off by the kidneys and render the urine alkaline.

According to the preceding hypothesis, the introduction of lactic acid or lactate of soda into the circulation would be followed by albuminuria, and M. Fourcault details some experiments which in his opinion prove the fact decisively. These experiments do not however appear to be of more than questionable value. The same remark applies to the comparison of the phenomena of cholera Asiatica with those of suppressed cutaneous excretion.

The next essay is a theory of chronic diseases. In this, which is simply the old doctrine of "catching cold," and "stopping of the pores," done up anew, M. Fourcault shows the origin of rickets, scrofula, phthisis, dropies, albuminuria, &c., from suppressed cutaneous secretion. The skin, to use his own expression, is the true key to pathology. The whole essay is a remarkable example of the power of a single idea in converging the most dissimilar facts towards itself. An essay on the fundamental principles and epochs of medicine concludes the first part.

*Hygiene of persons predisposed to chronic disease, and particularly to phthisis pulmonalis,* is the pith of the second part of M. Fourcault's volume. He devotes a chapter to a description of the incubation, or first period of phthisis, deriving his description of the symptoms from a translation by Lebeau of Sir James Clark's work on Pulmonary Consumption. The hygiene consists in a strenuous application of the single idea to practice. The patient is to be freely exposed to light and air; is to take sufficient and active exercise; to keep the skin clean and transpirable, &c. The chapter sets eloquently forth the advantages of physical education, and another advocates (what has been much neglected) the utility and advantage of selecting a trade or profession suited to the constitution.

"Every man of a lymphatic constitution and in consequence unfit for labours occupying his whole time, should take moderate gardening exercise. When a fatal hereditariness predisposes him to pulmonary consumption, he will fly from towns and devote himself to hunting, riding, or agriculture. If poor, he should enter the marine; if rich, travel a part of his life; if a landowner, he should be a farmer in climates where the air is dry, and on elevated ground where it is freely in motion. It cannot be too strongly recommended, that gardening is amongst the indispensable employments in hospitals, schools, orphan houses, and penitentiaries. The life of a soldier is not suited to persons predisposed to phthisis, for the military services render that disease prevalent among the soldiers, and particularly the infantry. Prudence and foresight consequently forbid the admission into the ranks of the army of young men, the sons of phthisical parents, or of those born in damp localities, or marshy plains; or of mechanics taken from cold and unhealthy workshops. It has been observed that a great number of the Flemish weavers enlisted into the Belgian army die of pulmonary phthisis. There can be no question that these different classes of recruits would escape fron this dangerous influence if they were entered for sea-service. This destination would be an immense benefit for themselves individually, and a considerable saving to the country." (p. 313-4.)

In the seventh chapter on *air and ventilation,* M. Fourcault states some interesting arguments to show the necessity of ventilating the skin. He would have hygienic precautions of this kind commence in infancy. The clothing of the infant in the cradle should be frequently changed, and the material of such a quality, that the air have free access to the cutaneous surface. When the temperature permits, he advises, that the infant be left for a short time every day completely naked.

One of the most interesting chapters in the book is that on gymnastics, containing a detailed description of Colonel Amoros' gymnastic establishment. The method of this distinguished gymnasiarch does not consist in directing this or that exercise, but in developing the faculties. An anthropobiological table ('tis M. Fourcault's expression) divides these faculties into as many divisions as there are principal branches of his method. Those purely physical are force, firmness, resistance, agility, velocity, address. The mixed or physico-moral are regularity, grace, zeal, courage, energy, perseverance. Among the faculties exclusively moral are prudence, foresight, temperance, generosity, and goodness. The muscular acts and gestures are regulated by chants or songs, and are therefore performed in rhythm. There are songs of labour, of loyalty, of emulation, benevolence, philanthropy, self-denial, &c.; each with *elementary* gestures and movements. These being done, the disciples of Amoros march for a time in column and platoon, and then disperse to a number of machines suitable to every form of gymnastic exercise. A highly-coloured description is given by our author, but allowing for this, there can be no doubt that the method of Amoros is well studied and strictly scientific, and ought to be known as well theoretically and practically by both heads of schools and medical practitioners. M. Fourcault states that the most morose children become gay and generous after a course of these exercises. The face of the lymphatic is animated, and loses its pale sallow tint; unnatural and morbid corpulency disappears; more healthy blood colours the skin; moderate exercise ceases to excite perspiration; in short, both the moral and physical condition of the individual are simultaneously changed. Children and delicate people should not be permitted to choose the kind of exercise they would prefer, because they will choose those in which a weak organ or limb is least exercised, whereas it is necessary to strengthen these by gymnastic movements.

M. Fourcault devotes a chapter to the hygienic effects of music and dancing, and urges the propriety of rendering it useful to the health as well as to the amusement. He suggests the separation of the sexes as the best mode of avoiding the voluptuous excitement of dancing.

The remaining chapters on repose and sleep, and excursions, and sea-voyages, sea-bathing, the use of mineral waters, and of pure water internally and externally, as well as of vapour-baths, contain a good deal of sound advice, and some minor points possess novelty, but not sufficient for our notice. A chapter on social hygiene recommends on various grounds that children should be taught and adults practise at least two employments, and that central, industrial, and agricultural *maisons de travail* (which can scarcely be termed *work*-houses in the English sense, though really such) should be established. These questions are not strictly within our province.

On a general review of M. Fourcault's volume, we find much more to approve than to condemn. It is discursive and illogical, but then it is amusing and instructive. The old-established maxim, insisting on air, exercise, and personal cleanliness, is enforced by new facts and arguments, not absolutely striking, but such that we willingly acknowledge our obligation to M. Fourcault for the useful information on a hackneyed subject we have acquired from his labours. Had he extended his physiological views of the influence of bodily exercise to the whole capillary system, instead of confining them to the skin, he would have come much nearer the truth; and, in this case, we are not sure but we would go along with him in most of his inferences both pathological and hygienic. At any rate, we have long been convinced, and have long acted on the conviction,—we humbly believe with unusual success,—that bodily exercise is one of the most important means in the cure of nearly all chronic diseases; and that it and proper diet and bathing, with a *modicum* of the simplest medicaments, will slowly yet surely restore lost health, in thousands of cases, where the ordinary routine of heroic medication, such as prevails so extensively in England, will not only fail to cure but inevitably augment the malady.

---

